# Multimorbidity and SARS-CoV-2–Related Outcomes: Analysis of a Cohort of Italian Patients

**DOI:** 10.2196/41404

**Published:** 2023-02-09

**Authors:** Alberto Catalano, Lucia Dansero, Winston Gilcrease, Alessandra Macciotta, Carlo Saugo, Luca Manfredi, Roberto Gnavi, Elena Strippoli, Nicolás Zengarini, Valeria Caramello, Giuseppe Costa, Carlotta Sacerdote, Fulvio Ricceri

**Affiliations:** 1 Centre for Biostatistics, Epidemiology, and Public Health Department of Clinical and Biological Sciences University of Turin Orbassano (TO) Italy; 2 UNESCO Chair in Sustainable Development and Territory Management University of Turin Turin Italy; 3 Klinik für Innere Medizin - Gastroenterologie, Hepatologie & Infektiologie Helios Klinikum Berlin-Buch Berlin Germany; 4 Unit of Epidemiology Regional Health Service ASLTO3 Grugliasco (TO) Italy; 5 Emergency Department and High Dependency Unit San Luigi Gonzaga University Hospital Orbassano (TO) Italy; 6 Unit of Cancer Epidemiology Città della Salute e della Scienza University Hospital Turin Italy

**Keywords:** multimorbidity, SARS-CoV-2, mortality, intensive care unit, epidemiology, COVID-19, pandemic, severity, cardiovascular, respiratory, disease, risk, public health, intervention

## Abstract

**Background:**

Since the outbreak of the COVID-19 pandemic, identifying the main risk factors has been imperative to properly manage the public health challenges that the pandemic exposes, such as organizing effective vaccination campaigns. In addition to gender and age, multimorbidity seems to be 1 of the predisposing factors coming out of many studies investigating the possible causes of increased susceptibility to SARS-CoV-2 infection and adverse outcomes. However, only a few studies conducted have used large samples.

**Objective:**

The objective is to evaluate the association between multimorbidity, the probability to be tested, susceptibility, and the severity of SARS-CoV-2 infection in the Piedmont population (Northern Italy, about 4 million inhabitants). For this purpose, we considered 5 main outcomes: access to the swab, positivity to SARS-CoV-2, hospitalization, intensive care unit (ICU) admission, and death within 30 days from the first positive swab.

**Methods:**

Data were obtained from different Piedmont health administrative databases. Subjects aged from 45 to 74 years and infections diagnosed from February to May 2020 were considered. Multimorbidity was defined both with the Charlson Comorbidity Index (CCI) and by identifying patients with previous comorbidities, such as diabetes and oncological, cardiovascular, and respiratory diseases. Multivariable logistic regression models (adjusted for age and month of infection and stratified by gender) were performed for each outcome. Analyses were also conducted by separating 2 age groups (45-59 and 60-74 years).

**Results:**

Of 1,918,549 subjects, 85,348 (4.4%) performed at least 1 swab, of whom 12,793 (14.9%) tested positive for SARS-CoV-2. Of these 12,793 subjects, 4644 (36.3%) were hospitalized, 1508 (11.8%) were admitted to the ICU, and 749 (5.9%) died within 30 days from the first positive swab. Individuals with a higher CCI had a higher probability of being swabbed but a lower probability of testing positive. We observed the same results when analyzing subjects with previous oncological and cardiovascular diseases. Moreover, especially in the youngest group, we identified a greater risk of being hospitalized and dying. Among comorbidities considered in the study, respiratory diseases seemed to be the most likely to increase the risk of having a positive swab and worse disease outcomes.

**Conclusions:**

Our study shows that patients with multimorbidity, although swabbed more frequently, are less likely to get infected with SARS-CoV-2, probably due to greater attention on protective methods. Moreover, a history of respiratory diseases is a risk factor for a worse prognosis of COVID-19. Nonetheless, whatever comorbidities affect the patients, a strong dose-response effect was observed between an increased CCI score and COVID-19 hospitalization, ICU admission, and death. These results are important in terms of public health because they help in identifying a group of subjects who are more prone to worse SARS-CoV-2 outcomes. This information is important for promoting targeted prevention and developing policies for the prioritization of public health interventions.

## Introduction

### Background

Since the outbreak of the COVID-19 pandemic in Italy and the world, identifying the risk factors for the development of a severe prognosis in patients with SARS-CoV-2 infection has been imperative to properly manage public health challenges that the pandemic exposes. For instance, organizing effective vaccination campaigns when just a limited number of vaccines were available was essential, and it is still important at the moment to decide on the prioritization of new campaigns for subsequent vaccine doses [1,2]. Moreover, the availability of accurate information about the possible evolution of the disease in the presence of risk factors can support the population in the choice of prudent behaviors aimed at preventing the spread of the contagion chain, with particular regard to the most vulnerable individuals.

Many studies in the literature have focused on the sociodemographic and clinical characteristics that are most associated with the development of a severe prognosis. Age and gender play an important role in determining the prognosis of patients with SARS-CoV-2. The elderly are especially at higher risk of becoming severely ill [3-5], and males are also a greater risk of a severe prognosis [5,6]. Among the several mechanisms proposed to explain the severity of COVID-19 in older adults, the increased burden of multimorbidity is 1 of the most relevant [7]. Moreover, several studies also have shown that multimorbidity affects COVID-19 severity in a way that is age independent, underscoring the need for an extensive study of the relationship between multimorbidity and SARS-CoV-2 outcomes [7].

Available evidence related to the identification of comorbidities that can negatively impact the prognosis in patients with SARS-CoV-2 refers essentially to observational, cross-sectional, retrospective cohort studies or case-control studies. There is general agreement on the role of hypertension, cardiovascular disease, and diabetes in increasing the risk of severe disease [5,6,8-10]. Therefore, comorbidities that affect the vascular system may play a key role in the worsening of clinical conditions in people affected by COVID-19. Moreover, there is some consensus on the role that obesity, cancer, chronic obstructive pulmonary disease (COPD), chronic kidney disease, and immunodepression could play in aggravating the prognosis [4-6,9]. However, these associations have been highlighted in a more sporadic and less uniform way.

In an initial phase, the correlation between the presence and the number of comorbidities with a more unfavorable outcome was pointed out to evaluate the prognosis of patients with SARS-CoV-2 [9]. This tool has been nevertheless effective and useful in individuating the key feature of the population at risk, which was crucial during the first phase of the pandemic.

It was in fact observed that subjects with some comorbidities (measured as a Charlson Comorbidity Index [CCI] [11]) equal to or greater than 1 are at higher risk of severe COVID-19 outcomes compared to subjects with no comorbidity [12,13]. Moreover, an innovative approach to the SARS-CoV-2 pandemic (the so-called “syndemic approach”) suggests that both bio-bio and biosocial interactions can act as complex drivers that increase subjects’ susceptibility to worsen COVID-19 outcomes [14-17].

Consequently, it is crucial to identify multifactorial profiles, including social determinants of health, and multimorbidity patterns associated with COVID-19 outcomes in order to recognize a broader syndemic health burden in specific subgroups of subjects with those characteristics [18,19].

### Study Design and Aim

This large population-based region-wide study based on administrative health databases aims to evaluate the association between multimorbidity and the susceptibility and severity of SARS-CoV-2 infection in the Piedmont population (Northern Italy, about 4 million inhabitants) aged from 45 to 74 years, considering COVID-19 infections diagnosed from February to May 2020.

Specifically, 5 different main outcomes were considered: access to the swab, positivity to SARS-CoV-2, hospitalization, admission to the intensive care unit (ICU), and death within 30 days from the first positive swab.

## Methods

### Study Population

For the analyses, data were obtained from the Piedmont Longitudinal Study (PLS), an administrative cohort based on the anonymous record linkage at the individual level of different social, health, and administrative databases. This study includes 2011 census data, hospital discharges, a mortality register, an outpatients register, an exemption register (in Italy, subjects with at least 1 chronic disease are exempt from paying for examinations related to their disease), and a drug prescriptions database. In addition, starting from February 2020, this study was enriched by the regional platform about COVID-19, in which infection data about subjects who had at least 1 contact with the regional health system related to SARS-CoV-2 are collected.

The study population comprised all assisted and domiciled subjects in Piedmont aged from 45 to 74 years. These 2 age classes were chosen in order to exclude young and elderly subjects, as they could have influenced the results. Furthermore, this choice made it possible to exclude the majority of patients residing in nursing homes in order to avoid any bias. With regard to SARS-CoV-2 infection, we considered those patients who developed the infection in Piedmont from February 22, 2020 (when the first case of SARS-CoV-2 was recorded in Italy), to May 31, 2020.

### Variables’ Definitions

Multimorbidity was defined using the CCI [11] retrieved by the record linkage with data of the hospital discharges and the drug prescription register between 2015 and 2019. In Multimedia Appendix 1, Table S1a, we present the algorithm’s definition.

The presence of prevalent oncological, cardiovascular, and respiratory diseases was also retrieved, as well as prevalent previous myocardial infarctions, heart failures, cerebrovascular diseases, and diabetes (algorithms presented in Multimedia Appendix 1, Table S1b).

Five different outcomes were considered: access to a SARS-CoV-2 test (nasal swab in almost all cases), positivity to the test, hospitalization, admission to the ICU, and mortality within 30 days after testing positive for SARS-CoV-2 infection. The 4 latter outcomes were compared to both the general population and to the total tested subjects or the subjects positive for SARS-CoV-2, as appropriate.

### Statistical Analysis

All the variables were described using absolute frequencies and percentages. For each outcome and exposure, we fitted a multivariable logistic regression model, in which the odds ratio (OR) estimates, together with their 95% Cls, were adjusted for age (used as a continuous variable) and for the month of infection or the first negative swab, where appropriate.

Moreover, to assess the probability of transition into various states on the basis of the CCI, multistate models were implemented. The possible transitions that were considered in the study involved 3 different states: from positivity to COVID-19 to hospitalization, from positivity to death within 30 days of the initial positive swab, and from hospitalization to 30-day mortality (Multimedia Appendix 1, Figure S1). In 1 case, the multistate models were stratified by the CCI score and adjusted for age and the month of infection; in the second case, the multistate models were adjusted for age, the month of infection, and the CCI.

All analyses were stratified by gender and 2 different age groups, 45-59 and 60-74 years, because we observed the differences between gender and age as effect modifiers.

Analyses were performed using SAS (V9.4) and R (V4.2.1).

### Ethical Considerations

All analyses were conducted according to the World Medical Association’s Declaration of Helsinki. In fact, the study is included in the National Statistical Plan and did not need approvals or permits from the ethics committee. For privacy purposes, the data used for analysis were anonymized.

## Results

### Study Population

Of 1,918,549 assisted and domiciled subjects in Piedmont aged from 45 to 74 years, 85,348 (4.4%) performed at least 1 swab during the observation period, of whom 12,793 (14.9%) tested positive for SARS-CoV-2. Of these 12,793 patients, 4644 (36.3%) were hospitalized due to COVID-19, 1508 (11.8%) were admitted to the ICU, and 749 (5.9%) died within 30 days from the first positive swab.

The descriptive statistics related to the exposures and variables used in the study are shown in Table 1. Among other results, the most interesting was related to gender. Overall, despite more women testing positive for SARS-CoV-2 (n=6832, 53.4%), higher percentages of men were hospitalized (3074/4644, 66.2%), were admitted to the ICU (1118/1508, 74.1%), and died (558/749, 74.5%). The same trend was observed when considering age: although more subjects aged 45-59 years tested positive for SARS-CoV-2 (n=7324, 57.2%), more individuals aged 60-74 years were hospitalized (2927/4644, 63%), were admitted to the ICU (1005/1508, 66.6%), and died (629/749, 84%).

Descriptive analyses also showed that multimorbidity (measured as CCI>1) increased the probability of being swabbed, hospitalized, admitted to the ICU, and dying from COVID-19.

Table 2 shows the OR estimates, together with their 95% Cls, related to accessing swabs for the entire population. The results showed that as the CCI increased, the likelihood of undergoing swab testing increased for both the male and the female population. In addition, other estimates indicated that being affected by 1 of the diseases considered in the study increased the likelihood of undergoing at least 1 swab test, especially in the case of heart failure, respiratory diseases, and cerebrovascular diseases (males aged 45-59 years: OR 2.60, 95% CI 2.30-2.94; males aged 60-74 years: OR 2.71, 95% CI 2.53-2.90; females aged 45-59 years: OR 1.63, 95% CI 1.45-1.85; females aged 60-74 years: OR 3.31, 95% CI 3.04-3.60).

The relationship between multimorbidity and SARS-CoV-2 positivity among those who performed at least 1 swab is shown in Table 3. The OR estimates indicated an inverse trend: the likelihood of testing positive for SARS-CoV-2 infection decreased as the CCI value increased. Moreover, those who were affected by oncological diseases had a significantly lower likelihood of testing positive for SARS-CoV-2, especially men (45-59 years old: OR 0.72, 95% CI 0.55-0.95; 60-74 years old: OR 0.63, 95% CI 0.53-0.73). Other specific diseases were less associated with the results of the SARS-CoV-2 infection test. On the contrary, when compared to the general population, multimorbid patients had an increased likelihood of testing positive (Multimedia Appendix 1, Table S2).

Table 4 shows the results related to the subjects who were admitted to the hospital from among those who tested positive for SARS-CoV-2. The results indicated that particularly for men from the ages of 45 to 59 years, the probability of hospital admission rose significantly as the CCI score increased (CCI=1: OR 1.50, 95% CI 1.20-1.88; CCI=2-3: OR 2.14, 95% CI 1.51-3.01; CCI=4+: OR 4.77, 95% CI 2.28-9.99). This also included females aged 60-74 years (CCI=1: OR 1.22, 95% CI 0.98-1,52; CCI=2-3: OR 2.28, 95% CI 1.74-2.99; CCI=4+: OR 4.22, 95% CI 2.56-6.97). Regarding the specific diseases considered in the study, the estimates suggested that except in the case of cerebrovascular diseases, patients affected by comorbidities had a significantly higher risk of being hospitalized. The results were similar when considering the entire population (Multimedia Appendix 1, Table S3).

The results related to admission to the ICU among those who tested positive with at least 1 swab are shown in Table 5. Unexpectedly, the association with multimorbidity was weak except for older women, while only a few associations were found with the specific diseases investigated. In contrast, compared to the general population, it emerged that patients with comorbidities and a higher CCI value had a higher risk of being admitted to intensive care for SARS-CoV-2 infection, especially in the case of respiratory diseases (Multimedia Appendix 1, Table S4).

Table 6 shows the OR estimates related to death within 30 days from the first positive swab among those who tested positive for SARS-CoV-2. Results suggested that as the CCI value increased, the risk of dying from COVID-19 significantly increased. When analyzing specific comorbidities, the estimates showed that the risk is significantly higher, especially among the younger population (eg, oncological diseases: OR 6.03, 95% CI 3.00-12.12 for males and OR 11.03, 95% CI 3.93-30.96 for females). Results were confirmed, considering the comparison with the general population (Multimedia Appendix 1, Table S5).

Table 7 and Multimedia Appendix 1, Figure S2, illustrate the outcomes of the multistate models. The probability of being hospitalized after testing positive for SARS-CoV-2 increased as the CCI increased, notably in the younger population, according to the hazard ratio (HR) estimates shown in the table. Furthermore, it appears that multimorbidity increased the risk of dying within 30 days of the first infection both without being hospitalized and after being hospitalized, with the exception of a small number of cases where some estimates were not statistically significant due to the low number of cases transiting among states.

Finally, stratifying the multistate models for the CCI revealed that both in the younger and the older population, the likelihood of COVID-19 severe outcomes considerably increased as the CCI score increased.

**Table 1:** Descriptive statistics related to the demographic and clinical characteristics of patients.

Variable and category		Entire population (N=1,918,549), n (%)	Access to the swab (n=85,348), n (%)	Positivity to SARS-Cov-2 (n=12,793), n (%)	Hospitalization (n=4644), n (%)	Admitted to the ICU^a^ (n=1508), n (%)	Death within 30 days (n=749), n (%)
**Gender**							
	Male	938,610 (48.9)	32,129 (37.6)	5961 (46.6)	3075 (66.2)	1118 (74.1)	558 (74.5)
	Female	979,939 (51.1)	53,219 (62.4)	6832 (53.4)	1569 (33.8)	390 (25.9)	191 (25.5)
**Age (years)**							
	45-59	1,062,861 (55.4)	54,672 (64.1)	7324 (57.2)	1717 (37.0)	503 (33.4)	120 (16.0)
	60-74	855,688 (44.6)	30,676 (35.9)	5469 (42.8)	2927 (63.0)	1005 (66.6)	629 (84.0)
**Month of first swab**							
	February-March	N/A^b^	14,134 (16.6)	5364 (41.9)	3088 (66.5)	1079 (71.5)	502 (67.0)
	April-May	N/A	71,214 (83.4)	7429 (58.1)	1556 (33.5)	429 (28.5)	247 (33.0)
**CCI^c^**							
	0	1,442,666 (75.2)	58,775 (68.8)	8,620 (67.4)	2,599 (56.0)	837 (55.5)	280 (37.4)
	1	335,685 (17.5)	16,180 (19.0)	2540 (19.8)	1091 (23.5)	361 (23.9)	175 (23.3)
	2-3	121,723 (6.3)	8099 (9.5)	1277 (10.0)	710 (15.3)	233 (15.5)	199 (26.6)
	4+	18,475 (1.0)	2294 (2.7)	356 (2.8)	244 (5.2)	77 (5.1)	95 (12.7)
Oncological disease		58,742 (3.1)	3993 (4.7)	525 (4.1)	303 (6.5)	83 (5.5)	82 (11.0)
Cardiovascular disease		136,335 (7.1)	9239 (10.8)	1480 (11.6)	797 (17.2)	252 (16.7)	217 (29.0)
Respiratory disease		50,328 (2.6)	4792 (5.6)	751 (5.9)	426 (9.2)	124 (8.2)	129 (17.2)
Myocardial infarction		40,288 (2.1)	2416 (2.8)	420 (3.3)	278 (6.0)	99 (6.6)	78 (10.4)
Heart failure		7,817 (0.4)	762 (0.9)	117 (0.9)	79 (1.7)	22 (1.5)	28 (3.7)
Cerebrovascular disease		23,753 (1.2)	2197 (2.6)	378 (2.9)	186 (4.0)	53 (3.5)	72 (9.6)
Diabetes		157,214 (8.2)	8335 (9.8)	1547 (12.1)	896 (19.3)	352 (23.3)	239 (31.9)

^a^ICU: intensive care unit.

^b^N/A: not applicable.

^c^CCI: Charlson Comorbidity Index.

**Table 2:** OR^a^ estimates related to access to the swab for the entire population (N=85,348), stratified by gender and age group (1 model for each variable).

Variable and category		Male		Female	
		Age 45-59 years (n=16,978, 19.9%), OR (95% CI)^b^	Age 60-74 years (n=15,151, 17.8%), OR (95% CI)^b^	Age 45-59 years (n=37,694, 44.2%), OR (95% CI)^b^	Age 60-74 years (n=15,525, 18.1%), OR (95% CI)^b^
**CCI^c^**					
	0	Reference	Reference	Reference	Reference
	1	1.37 (1.32-1.43)	1.31 (1.26-1.37)	1.22 (1.19-1.26)	1.39 (1.34-1.45)
	2-3	2.41 (2.26-2.56)	2.31 (2.21-2.42)	1.37 (1.31-1.45)	2.19 (2.09-2.30)
	4+	4.41 (3.93-4.95)	4.93 (4.62-5.26)	2.22 (1.91-2.57)	5.18 (4.73-5.68)
Oncological disease		2.31 (2.10-2.53)	2.04 (1.93-2.15)	1.18 (1.10-1.27)	1.80 (1.69-1.92)
Cardiovascular disease		1.94 (1.84-2.05)	2.11 (2.03-2.19)	1.45 (1.37-1.52)	2.19 (2.09-2.29)
Respiratory disease		2.41 (2.24-2.59)	3.20 (3.03-3.37)	1.68 (1.57-1.79)	3.34 (3.13-3.55)
Myocardial infarction		1.57 (1.42-1.74)	1.71 (1.62-1.81)	1.53 (1.32-1.77)	1.94 (1.76-2.14)
Heart failure		2.45 (2.00-3.00)	3.07 (2.78-3.40)	1.71 (1.27-2.29)	3.28 (2.81-3.83)
Cerebrovascular disease		2.60 (2.30-2.94)	2.71 (2.53-2.90)	1.63 (1.45-1.85)	3.31 (3.04-3.60)
Diabetes		1.46 (1.37-1.55)	1.46 (1.40-1.52)	1.21 (1.15-1.27)	1.60 (1.52-1.67)

^a^OR: odds ratio.

^b^Estimates adjusted for age. All estimates (95% confidence level) were significant.

^c^CCI: Charlson Comorbidity Index.

**Table 3:** OR^a^ estimates related to positivity to SARS-CoV-2 among those who performed at least 1 swab (N=12,793), stratified by gender and age group (1 model for each variable).

Variable and category		Male		Female	
		Age 45-59 years (n=2847, 22.3%), OR (95% CI)^b^	Age 60-74 years (n=3114, 24.3%), OR (95% CI)^b^	Age 45-59 years (n=4477, 35.0%), OR (95% CI)^b^	Age 60-74 years (n=2355, 18.4%), OR (95% CI)^b^
**CCI^c^**					
	0	Reference	Reference	Reference	Reference
	1	0.97 (0.87-1.10)	1.07 (0.96-1.19)	1.01 (0.93-1.10)	1.05 (0.94-1.17)
	2-3	0.79 (0.66-0.95)^d^	0.86 (0.77-0.97)^d^	0.98 (0.84-1.14)	0.93 (0.81-1.07)
	4+	0.60 (0.42-0.86)^d^	0.79 (0.67-0.95)^d^	0.67 (0.41-1.09)	0.91 (0.71-1.17)
Oncological disease		0.72 (0.55-0.95)^d^	0.63 (0.53-0.73)^d^	0.82 (0.66-1.02)	0.70 (0.57-0.87)^d^
Cardiovascular disease		0.80 (0.68-0.94)^d^	0.91 (0.82-1.01)	0.89 (0.77-1.04)	1.02 (0.89-1.16)
Respiratory disease		0.89 (0.73-1.09)	0.88 (0.76-1.01)	0.82 (0.67-1.02)	1.05 (0.89-1.25)
Myocardial infarction		0.83 (0.62-1.12)	0.84 (0.72-0.98)	0.58 (0.34-0.99)^d^	1.10 (0.84-1.43)
Heart failure		0.46 (0.22-0.98)^d^	0.78 (0.59-1.04)	0.84 (0.33-2.14)	1.10 (0.74-1.65)
Cerebrovascular disease		0.95 (0.67-1.35)	1.18 (0.99-1.41)	0.83 (0.56-1.22)	1.12 (0.90-1.41)
Diabetes		1.02 (0.86-1.20)	1.25 (1.13-1.39)^d^	1.16 (1.01-1.34)^d^	1.11 (0.98-1.27)

^a^OR: odds ratio.

^b^Estimates adjusted for age and the month of the first swab.

^c^CCI: Charlson Comorbidity Index.

^d^Significant estimates (95% confidence level).

**Table 4:** OR^a^ estimates related to admission to the hospital among those who tested positive for SARS-CoV-2 (N=4644), stratified by gender and age group (1 model for each variable).

Variable and category		Male		Female	
		Age 45-59 years (n=1101, 23.7%), OR (95% CI)^b^	Age 60-74 years (n=1974, 42.5%), OR (95% CI)^b^	Age 45-59 years (n=616, 13.3%), OR (95% CI)^b^	Age 60-74 years (n=953, 20.5%), OR (95% CI)^b^
**CCI^c^**					
	0	Reference	Reference	Reference	Reference
	1	1.50 (1.20-1.88)^d^	1.51 (1.23-1.84)^d^	1.50 (1.20-1.88)^d^	1.22 (0.98-1.52)
	2-3	2.14 (1.51-3.01)^d^	1.40 (1.12-1.75)^d^	2.49 (1.77-3.50)^d^	2.28 (1.74-2.99)^d^
	4+	4.77 (2.28-9.99)^d^	1.98 (1.41-2.78)^d^	2.45 (0.86-6.94)	4.22 (2.56-6.97)^d^
Oncological disease		2.62 (1.52-4.51)^d^	1.27 (0.93-1.73)	2.26 (1.39-3.68)^d^	1.97 (1.34-2.92)^d^
Cardiovascular disease		1.53 (1.13-2.08)^d^	1.30 (1.01-1.49)^d^	1.69 (1.17-2.44)^d^	1.52 (1.19-1.95)^d^
Respiratory disease		2.17 (1.48-3.19)^d^	1.47 (1.12-1.92)^d^	2.13 (1.31-3.45)^d^	2.16 (1.57-2.96)^d^
Myocardial infarction		1.84 (1.03-3.30)^d^	1.47 (1.09-1.97)^d^	2.05 (0.61-6.94)	2.36 (1.44-3.89)^d^
Heart failure		7.90 (1.53-40.70)^d^	2.18 (1.23-3.86)^d^	1.25 (0.12-12.56)	1.88 (0.90-3.93)
Cerebrovascular disease		1.39 (0.69-2.81)	0.92 (0.68-1.26)	1.30 (0.47-3.63)	1.11 (0.73-1.71)
Diabetes		1.65 (1.20-2.28)^d^	1.61 (1.31-1.96)^d^	1.89 (1.35-2.63)^d^	2.10 (1.64-2.69)^d^

^a^OR: odds ratio.

^b^Estimates adjusted for age and the month of the first swab.

^c^CCI: Charlson Comorbidity Index.

^d^Significant estimates (95% confidence level).

**Table 5:** OR^a^ estimates related to admission to the ICU^b^ among those who tested positive for SARS-CoV-2 (N=1508), stratified by gender and age group (1 model for each variable).

Variable and category		Male		Female	
		Age 45-59 years (n=370, 24.5%), OR (95% CI)^c^	Age 60-74 years (n=748, 49.6%), OR (95% CI)^c^	Age 45-59 years (n=133, 8.8%), OR (95% CI)^c^	Age 60-74 years (n=257, 17.1%), OR (95% CI)^c^
**CCI^d^**					
	0	Reference	Reference	Reference	Reference
	1	1.39 (1.04-1.86)^e^	1.15 (0.94-1.42)	1.06 (0.66-1.70)	1.25 (0.91-1.73)
	2-3	1.32 (0.85-2.05)	1.04 (0.82-1.32)	2.46 (1.39-4.36)^e^	1.89 (1.32-2.71)^e^
	4+	1.91 (0.83-4.39)	1.09 (0.77-1.54)	1.33 (0.17-10.35)	2.35 (1.32-4.19)^e^
Oncological disease		1.44 (0.74-2.80)	0.86 (0.62-1.20)	0.91 (0.28-2.94)	0.97 (0.55-1.71)
Cardiovascular disease		1.58 (1.09-2.30)^e^	0.84 (0.68-1.03)	2.02 (1.08-3.76)^e^	1.23 (0.87-1.75)
Respiratory disease		1.53 (0.96-2.45)	0.84 (0.63-1.14)	1.92 (0.81-4.51)	1.53 (1.01-2.32)^e^
Myocardial infarction		2.05 (1.08-3.86)^e^	1.04 (0.77-1.40)	2.00 (0.25-15.85)	1.75 (0.95-3.22)
Heart failure		N/A^f^	1.16 (0.66-2.06)	N/A	1.39 (0.52-3.69)
Cerebrovascular disease		1.39 (0.55-3.50)	0.74 (0.50-1.08)	1.08 (0.14-8.21)	0.94 (0.49-1.80)
Diabetes		1.43 (0.98-2.11)	1.58 (1.31-1.92)^e^	2.58 (1.51-4.42)^e^	2.14 (1.57-2.92)^e^

^a^OR: odds ratio.

^b^ICU: intensive care unit.

^c^Estimates adjusted for age and the month of the first swab.

^d^CCI: Charlson Comorbidity Index.

^e^Significant estimates (95% confidence level).

^f^N/A: not applicable.

**Table 6:** OR^a^ estimates related to death within 30 days from the first positive swab among those who tested positive to SARS-CoV-2 (N=749), stratified by gender and age group (1 model for each variable).

Variable and category		Male		Female	
		Age 45-59 years (n=370, 24.5%), OR (95% CI)^b^	Age 60-74 years (n=748, 49.6%), OR (95% CI)^b^	Age 45-59 years (n=133, 8.8%), OR (95% CI)^b^	Age 60-74 years (n=257, 17.1%), OR (95% CI)^b^
**CCI^c^**					
	0	Reference	Reference	Reference	Reference
	1	1.56 (0.89-2.75)	1.33 (1.02-1.74)^d^	2.26 (0.68-7.56)	1.50 (0.99-2.27)
	2-3	5.33 (3.06-9.27)^d^	2.24 (1.72-2.91)^d^	16.80 (6.35-44.45)^d^	2.43 (1.58-3.73)^d^
	4+	17.32 (7.91-37.90)^d^	3.26 (2.29-4.64)^d^	31.88 (5.96-170.43)^d^	5.90 (3.31-10.52)^d^
Oncological disease		6.03 (3.00-12.12)^d^	1.46 (1.04-2.05)^d^	11.03 (3.93-30.96)^d^	1.38 (0.77-2.47)
Cardiovascular disease		3.52 (2.12-5.86)^d^	1.56 (1.24-1.95)^d^	2.81 (0.82-9.62)	2.04(1.41-2.94)^d^
Respiratory disease		3.89 (2.16-7.02)^d^	2.07 (1.54-2.78)^d^	5.88 (1.70-20.39)^d^	2.92 (1.91-4.45)^d^
Myocardial infarction		2.80 (1.15-6.81)^d^	1.50 (1.09-2.06)^d^	N/A^e^	2.16 (1.41-2.94)^d^
Heart failure		8.42 (1.66-42.77)^d^	1.79 (0.99-3.23)	N/A	6.04 (2.70-13.53)^d^
Cerebrovascular disease		5.68 (2.24-14.36)^d^	1.96 (1.38-2.79)^d^	N/A	2.06 (1.17-3.65)^d^
Diabetes		2.36 (1.34-4.15)^d^	1.79 (1.43-2.23)^d^	5.72 (2.21-14.79)^d^	2.35 (1.64-3.35)^d^

^a^OR: odds ratio.

^b^Estimates adjusted for age and the month of the first swab.

^c^CCI: Charlson Comorbidity Index.

^d^Significant estimates (95% confidence level).

^e^N/A: not applicable.

**Table 7:** HR^a^ estimates related to multistate models for each possible transition, stratified by gender and age group.

CCI^b^		Male (age 45-59 years), HR (95% CI)^c^	Male (age 60-74 years), HR (95% CI)^c^	Female (age 45-59 years), HR (95% CI)^c^	Female (age 60-74 years), HR (95% CI)^c^
**Positive --> hospitalization**					
	0	Reference	Reference	Reference	Reference
	1	1.34 (1.15-1.56)	1.19 (1.06-1.33)	1.43 (1.17-1.75)	1.19 (1.01-1.39)
	2-3	1.48 (1.18-1.86)	1.09 (0.96-1.23)	2.49 (1.90-3.26)	1.61 (1.34-1.93)
	4+	2.71 (1.85-3.97)	1.25 (1.04-1.49)	2.42 (1.09.5.33)	2.39 (1.81-3.16)
**Positive --> death within 30 days from first infection**					
	0	Reference	Reference	Reference	Reference
	1	3.87 (0.01-infinity)	0.73 (0.29-1.87)	N/A^d^	2.67 (1.05-6.80)
	2-3	1.20 (0.01-infinity)	2.10 (1.03-4.28)	22.57 (2.32-219.69)	4.13 (1.53-11.12)
	4+	220750.22 (0.01-infinity)	3.33 (1.37-8.11)	N/A	7.69 (2.00-29.52)
**Hospitalization --> death within 30 days from first infection**					
	0	Reference	Reference	Reference	Reference
	1	1.42 (0.30-6.61)	1.22 (0.94-1.59)	8.29 (2.81-24.38)	1.40 (0.89-2.21)
	2-3	5.10 (1.34-19.44)	1.92 (1.49-2.49)	0.03 (0.01-37674.39)	1.61 (0.99-2.61)
	4+	728.13 (355.06-1493.24)	2.38 (1.71-3.31)	0.41 (0.01-93403.78)	3.46 (1.98-6.04)

^a^HR: hazard ratio.

^b^CCI: Charlson Comorbidity Index.

^c^Estimated adjusted for age and the month of the first swab.

^d^N/A: not applicable.

## Discussion

### Principal Findings

In this study, we analyzed the association between multimorbidity and SARS-CoV-2 outcomes in the population of the large Italian region of Piedmont. It emerged that multimorbidity is a strong risk factor for a worse prognosis of COVID-19, especially in the younger population. In addition, results highlighted that although subjects with previous diseases were more likely to be swabbed, they had a general lower risk of being infected.

Regarding access to the swab during the first wave of the COVID-19 pandemic, the estimates showed that the likelihood of being swabbed was greater for patients with previous diseases, regardless of the kind of disease, and this risk increased with the increase in multimorbidity (measured as the CCI). This is consistent with what was observed during the outbreak of the COVID-19 pandemic in early 2020, when only few laboratories were equipped for performing the SARS-CoV-2 test from nasal swabs and the Italian government published strict clinical and epidemiological criteria for accessing the tests (among those, subjects with a chronic disease are considered at higher risk), which were also limited in availability [20]. In addition, from April 2020, patients with chronic diseases were swabbed to access outpatient services, such as dialysis or cancer treatments.

Focusing on positivity to SARS-CoV-2, when considering the entire population, it emerged that the risk of infection is higher for subjects with previous comorbidities, in line with other studies [21,22]. This occurs because subjects affected by comorbidities, due to poor clinical conditions, generally perform more swabs compared to healthy subjects; thus, their probability of testing positive for COVID-19 is higher. In contrast, when investigating the likelihood of testing positive only among those who performed at least 1 swab, we identified that patients with comorbidities are less likely to test positive for SARS-CoV-2. This result could be due to the fact that subjects with poorer clinical conditions pay more attention to protective methods, such as social distancing, wearing masks, handwashing, and avoiding overcrowded places, which significantly reduce the risk of SARS-CoV-2 infection [23].

In our investigation of hospitalization, we also found that multimorbidity, which in our study was measured through the CCI, is a strong risk factor for a worse prognosis of COVID-19. This is consistent with a number of studies conducted on the topic [12,13,24].

Among all possible comorbidities, Chudasama et al [24] identified in their study that pre-existing hypertension is the most prevalent condition in subjects affected by severe SARS-CoV-2 infection, and it mainly coexists with other previous comorbidities: stroke, diabetes, and chronic kidney disease. However, the risk of severe COVID-19 is highest in patients affected by both previous diabetes and pre-existing chronic kidney disease. One of the possible reasons patients with multiple comorbidities have an increased risk of developing severe SARS-CoV-2 is that they generally use inhibitors of the renin-angiotensin system (RAS) to limit the effect of their comorbidities. These inhibitors cause the overexpression of angiotensin-converting enzyme 2 (ACE2), which in turn facilitates the entry of SARS-CoV-2 into human target cells. In addition, high levels of some biomarkers, such as C-reactive protein, D-dimer, procalcitonin, and ferritin, in individuals with multiple comorbidities may lead or contribute to a worse prognosis of COVID-19 [13]. In fact, it has been found that these biomarkers are often elevated in subjects who contract severe infection.

In our study, we also identified that the risk of being admitted to the ICU among those who tested positive to the virus was not significantly higher for subjects affected by multimorbidity, except in a few cases, such as cardiovascular diseases and diabetes. Given the findings that emerged from our research in relation to hospital admissions and death, this result would seem to be counterintuitive. This finding may be attributable to the decisions made by health care professionals during the first pandemic wave about which patients to admit to intensive care and which not to admit. In fact, health care professionals had to choose which subjects to admit to the ICU on the basis of their clinical conditions, the number and severity of comorbidities, age, and possible benefits of admission, also due in the peak weeks of the pandemic to the limited number of beds available. Only subjects with the highest clinical outcomes and potential benefits were therefore admitted to the ICU. The mortality data support this explanation; in fact, as expected, the probability of dying from COVID-19 increased dramatically as the CCI value increased.

Considering specific comorbidities, we found that subjects with almost all previous comorbidities have a greater risk of developing a worse prognosis of COVID-19, considering both the general population and only patients with SARS-CoV-2. This is in line with other studies that have highlighted this topic. According to some research that investigated the relationship between oncological diseases and SARS-CoV-2–related outcomes [25-27], people with cancer have a greater risk of developing a worse prognosis of COVID-19. This finding could be due to the fact that patients with cancer are particularly susceptible to the immunosuppressive state caused by antitumor therapies received, such as radiotherapy and chemotherapy [26]. In addition, the risk of COVID-19 severity has been found to be higher for individuals who received their last chemotherapy within 14 days of admission [28].

Further studies that have investigated the association between cardiovascular or cerebrovascular diseases and COVID-19 outcomes found that the risk of severe SARS-CoV-2 is significantly higher for subjects affected by these comorbidities [8,29]. Furthermore, according to a meta-analysis conducted on 56 studies [30], it was shown that the risk of developing severe COVID-19 is greater, considering patients with SARS-CoV-2 and any pre-existing cardiovascular diseases and also when considering specific cardiovascular comorbidities separately, such as acute cardiac injury or heart failure, as shown in our study. This association could be due to the fact that drugs used to limit cardiovascular and cerebrovascular risk, such as ACE inhibitors and angiotensin II receptor blockers (ARBs), have numerous effects that could influence the susceptibility to or the severity of COVID-19. In fact, it was demonstrated that ACE inhibitors and ARBs increase the expression of ACE2, which is the viral receptor for SARS-COV-2 and facilitates the virus entry into pneumocytes [29,31].

In relation to diabetes, several studies have also shown that it is a risk factor for the mortality and severity of COVID-19. In fact, according to different studies and meta-analyses [32-35], the risk of contracting severe SARS-CoV-2 or dying from the virus infection has been found to be significantly higher for patients with diabetes. One possible reason for this is that subjects with diabetes have a higher risk of uncontrolled inflammatory response, higher levels of tissue injury–related enzymes, a higher hypercoagulable state, and higher serum levels of inflammatory biomarkers, such as C-reactive protein, D-dimer, interleukin-6 (IL-6), serum ferritin, and coagulation index. This greater susceptibility to an inflammatory status could lead to a worse prognosis of COVID-19, especially in patients with poor glycemic control, since hyperglycemia is a powerful antagonist of the immune response [32,36]. In addition, these subjects also have an immune system downregulated by impairing the function of innate immunity, such as chemotaxis and the activity of neutrophils and macrophages, that could lead to severe COVID-19 outcomes or mortality [35].

Other studies have instead found a relationship between pre-existing respiratory comorbidities and the risk of developing a worse prognosis of COVID-19, especially in the case of COPD, asthma, and obstructive sleep apnea (OSA) [37-39]. This association, on the one hand, is due to the fact that previous respiratory diseases could worsen lung function, could make the airways hypersensitive, and could cause immune alteration in the patients, possibly leading to subjects contracting more severe SARS-CoV-2 [38]. On the other hand, especially in the case of pre-existing OSA, hypercapnia and hypoxemia, surges of sympathetic activation, and increased inflammatory markers may contribute to contracting more severe SARS-CoV-2 [39].

Although in our study, we only investigated diabetes and oncological, cardiovascular, cerebrovascular, and respiratory diseases, previous research has shown that other comorbidities, such as chronic liver and chronic kidney diseases, are also associated with a more severe prognosis of COVID-19 [40,41].

### Strengths and Limitations

This study represents an advance on what is already present in the literature. In fact, compared to what has been investigated on this topic to date, in this research, (1) a population study was conducted instead of a clinical study, which made it possible both to have a much larger number of people available and to investigate what happened in an entire region and not only in hospitals or health care institutions, whose studies are generally conducted in more advanced facilities (eg, university hospitals); (2) the period analyzed in the study (ie, the first wave of the COVID-19 pandemic) made it possible to obtain results that are not influenced by the various organized prevention/vaccination strategies implemented subsequently; and (3) the assessment of the probability to be tested could be a bias in susceptibility evaluation.

The main limitation of this study is that data derived from record linkage of health administrative databases, where information bias (although not differential) is present, and depth of information were limited. A further limitation is that data were based only on the first wave of the pandemic. Moreover, the administrative nature of the sample was not able to capture the social elements that could be used to fully conceive a syndemic approach; however, the obtained results identified a disease-disease interaction that could be the basis for further research in this framework.

### Conclusion

In a sample of nearly 2 million subjects, our study is 1 of the first to assess the association between multimorbidity and all SARS-CoV-2–related outcomes. Our findings show that during the first wave of the pandemic, patients with multimorbidity were closely monitored, as proven by a high frequency of tests for SARS-CoV-2. However, although swabbed more frequently, they appeared to be less likely to become infected with SARS-CoV-2, probably due to greater attention paid to protective methods. However, a history of respiratory diseases is a risk factor for a worse prognosis of COVID-19. Nonetheless, whatever comorbidities affect the patients, a strong dose-response effect was observed between an increased CCI score and COVID-19 hospitalization, ICU admission, and death.

These results are critical to public health policy and planning as they help in identifying a group of subjects who are more prone to worse SARS-CoV-2 outcomes. This information is particularly important for the current pandemic scenario, where the emergency has given way to the SARS-CoV-2 pandemic's embeddedness into daily life. In fact, these results suggest that future clinical and public health interventions (eg, vaccination prioritization, early monoclonal antibody treatment, prevention measurement and campaigns) should be centered on the multimorbid patient category because they are more likely to need to be protected from COVID-19 severe outcomes. Furthermore, the early response to the COVID-19 pandemic provided a framework for our observations that might be applicable to future health challenges. It will be crucial for future research to investigate the biosocial relationship in this context.

## Appendix

Multimedia Appendix 1 Supplementary material.

## Abbreviations

ACE: angiotensin-converting enzyme
ARB: angiotensin II receptor blocker
CCI: Charlson Comorbidity Index
COPD: chronic obstructive pulmonary disease
HR: hazard ratio
ICU: intensive care unit
OR: odds ratio
OSA: obstructive sleep apnea

## Data Availability

Raw data cannot be made freely available because of restrictions imposed by the ethical committees, which do not allow open/public sharing of data on individuals. However, aggregated data are available for other researchers upon request to the corresponding author.
