# Supplementary material for: Multimorbidity and SARS-CoV-2–Related Outcomes: Analysis of a Cohort of Italian Patients
Source: JMIR Public Health Surveill. 2023 Feb 9;9:e41404. doi: 10.2196/41404 (PMC9951075; doi:10.2196/41404)

**Multimedia Appendix 1**

**Table S1**

**a)** **Algorithm related to the Charlson Comorbidity Index (CCI) definition**

| **Disease** | **Weight** | **Hospital Discharges Codes^a^** | **Drug prescriptions Codes^b^** | **Disease exemptions Codes** |
| --- | --- | --- | --- | --- |
| **Charlson Comorbidity Index (CCI)** | | | | |
| ***Diabetes*** | 1 | 250 | A10 | 013 |
| ***Myocardial infarction*** | 1 | 410-414 | - | - |
| ***Peripheral hearth disease*** | 1 | 440-448 | - | - |
| ***Congestive heart failure*** | 1 | 398.91^c^, 402^c^, 404.01^c^, 404.03^c^, 404.11^c^, 404.13^c^, 404.91^c^, 404.93^c^,  425.4-425.5^c^, 425.9^c^, 428^c^, 785.50^-^785.51^c^ | - | - |
| ***Cerebrovascular disease*** | 1 | 430-434, 436-438 | - | 0B02 |
| ***Dementia*** | 1 | 046, 290-292,  294, 331 | - | N06D |
| ***Mild liver disease*** | 1 | 070 | - | 016 |
| ***Hemiplegia*** | 2 | 342, 436 | - | - |
| ***Oncological disease*** | 2 | 140-208 | - | - |
| ***Ulcer*** | 1 | 531-534 | - | - |
| ***Severe liver disease*** | 3 | 571 | - | 008 |
| ***Connective tissue disease*** | 1 | 696.0, 710.0,  714.0, 720.0 | - | 006, 028,  030, 045, 054, |
| ***Moderate or severe kidney disease*** | 2 | 250.4, 403-404,  582-583, 585-588,  590.0, 753.1, V42.0,  V56; 38.95^d^, 39.27^d^, 39.42^d^, 39.43^d^, 39.95^d^, 54.93^d^, 54.98^d^, 55.23^d^, 55.6^d^ | B03XA01, B03XA02, B03XA03, V03AE01, V03AE02, V03AE03,  V03AE05 | 023 |
| ***Chronic pulmonary disease*** | 1 | 518.81^c^, 518.84^c^, 786.0^c^, 786.2^c^, 786.4^c^; 490-494, 496 | R03A, R03CC02, R03CC03, R03CC04, R03CK, R03BB01, R03BB02, R03BB04, R03DA01, R03DA04, R03DA05, R03DA08, RO3DA11, R03DA49 | 024 |

^a^ International Classification of Diseases – IX
 ^b^ Anatomical Therapeutic Chemical 7 Classification
 ^c^ Only main diagnosis
 ^d^ Intervention procedures

| **Disease** | **Hospital Discharges Codes^a^** | **Drug prescriptions Codes^b^** | **Disease exemptions Codes** |
| --- | --- | --- | --- |
| **Specific Disease** | | | |
| ***Oncological disease*** | 140-208 | - | - |
| ***Cardiovascular disease*** | 390-459 | - | - |
| ***Respiratory disease*** | 460-519 | - | - |
| ***Cerebrovascular disease*** | 430-434, 436-438 | - | 0B02 |
| ***Diabetes*** | 250 | A10 | 013 |
| ***Myocardial infarction*** | 410-414 | - | - |
| ***Congestive heart failure*** | 398.91^c^, 402^c^, 404.01^c^, 404.03^c^, 404.11^c^, 404.13^c^, 404.91^c^, 404.93^c^,  425.4-425.5^c^, 425.9^c^, 428^c^,  785.50^-^785.51^c^ | **-** | **-** |

**b**) **Algorithm related to the definition of the specific diseases considered in the study**

^a^ International Classification of Diseases – IX
^b^ Anatomical Therapeutic Chemical 7 Classification
^c^ Only main diagnosis

**Table S2: Odds ratio (OR) estimates related to positivity to SARS-CoV-2 on entire population, stratified by gender and age group (one model for each variable)**

|  |  | **Male** | | **Female** | |
| --- | --- | --- | --- | --- | --- |
| **Variable** | **Category** | ***(N=2,847)* 45-59 ___________  OR (95% CI)^a^** | ***(N=3,114)* 60-74 ___________  OR (95% CI)^a^** | ***(N=4,477)* 45-59 ___________  OR (95% CI)^a^** | ***(N=2,355)* 60-74**  **___________  OR (95% CI)^a^** |
| **Charlson**  **Comorbidity  Index** | 0 | Ref | Ref | Ref | Ref |
|  | 1 | **1.28 (1.16-1.42)** | **1.32 (1.21-1.44)** | **1.21 (1.11-1.30)** | **1.43 (1.30-1.58)** |
|  | 2-3 | **1.93 (1.64-2.25)** | **1.95 (1.77-2.15)** | **1.32 (1.15-1.52)** | **2.09 (1.85-2.36)** |
|  | 4+ | **2.56 (1.85-3.53)** | **3.68 (3.18-4.26)** | 1.48 (0.93-2.36) | **4.63 (3.72-5.77)** |
| **Oncological**  **disease** | - | **1.80 (1.41-2.29)** | **1.39 (1.22-1.59)** | 0.97 (0.79-1.20) | **1.36 (1.14-1.63)** |
| **Cardiovascular disease** | - | **1.53 (1.33-1.76)** | **1.83 (1.68-1.99)** | **1.30 (1.13-1.50)** | **2.15 (1.92-2.41)** |
| **Respiratory**  **disease** | - | **2.12 (1.79-2.53)** | **2.56 (2.27-2.87)** | **1.37 (1.12-1.68)** | **3.38 (2.92-3.91)** |
| **Myocardial**  **infarction** | - | 1.26 (0.97-1.64) | **1.56 (1.38-1.77)** | 0.90 (0.54-1.50) | **2.14 (1.71-2.69)** |
| **Heart failure** | - | 1.20 (0.62-2.32) | **2.23 (1.75-2.83)** | 1.35 (0.56-3.27) | **3.46 (2.45-4.90)** |
| **Cerebrovascular disease** | - | **1.99 (1.45-2.74)** | **2.53 (2.19-2.92)** | 1.31 (0.91-1.90) | **3.23 (2.66-3.94)** |
| **Diabetes** | - | **1.43 (1.23-1.65)** | **1.63 (1.50-1.77)** | **1.36 (1.19-1.55)** | **1.70 (1.52-1.90)** |

^a^ Estimates adjusted for age

Significant estimates (95% confidence level) are shown in bold type

**Table S3: Odds ratio (OR) estimates related to admission to hospital on entire population, stratified by gender and age group (one model for each variable)**

|  |  | **Male** | | **Female** | |
| --- | --- | --- | --- | --- | --- |
| **Variable** | **Category** | ***(N=1,101)* 45-59 ___________  OR (95% CI)^a^** | ***(N=1,974)* 60-74 ___________  OR (95% CI)^a^** | ***(N=616)* 45-59 ___________  OR (95% CI)^a^** | ***(N=953)* 60-74**  **___________  OR (95% CI)^a^** |
| **Charlson**  **Comorbidity  Index** | 0 | Ref | Ref | Ref | Ref |
|  | 1 | **1.58 (1.36-1.84)** | **1.50 (1.34-1.67)** | **1.68 (1.38-2.05)** | **1.64 (1.40-1.92)** |
|  | 2-3 | **2.71 (2.18-3.38)** | **2.11 (1.87-2.39)** | **2.75 (2.08-3.63)** | **3.20 (2.70-3.80)** |
|  | 4+ | **4.55 (3.07-6.75)** | **4.25 (3.57-5.06)** | **3.73 (1.66-8.37)** | **8.35 (6.36-10.95)** |
| **Oncological**  **disease** | - | **2.55 (1.85-3.51)** | **1.48 (1.26-1.74)** | **1.94 (1.31-2.87)** | **1.91 (1.51-2.42)** |
| **Cardiovascular disease** | - | **1.84 (1.50-2.25)** | **1.86 (1.68-2.07)** | **2.04 (1.50-2.79)** | **2.60 (2.21-3.06)** |
| **Respiratory**  **disease** | - | **3.03 (2.39-3.83)** | **2.64 (2.28-3.05)** | **2.61 (1.76-3.87)** | **4.70 (3.86-5.71)** |
| **Myocardial**  **infarction** | - | **1.69 (1.19-2.41)** | **1.74 (1.50-2.03)** | 1.57 (0.59-4.21) | **3.03 (2.26-4.05)** |
| **Heart failure** | - | **2.27 (1.07-4.78)** | **2.54 (1.92-3.35)** | 1.77 (0.25-12.59) | **4.37 (2.76-6.89)** |
| **Cerebrovascular disease** | - | **1.97 (1.20-3.23)** | **2.20 (1.82-2.65)** | 1.54 (0.64-3.72) | **3.22 (2.39-4.32)** |
| **Diabetes** | - | **1.87 (1.53-2.29)** | **1.85 (1.67-2.05)** | **2.32 (1.75-3.07)** | **2.35 (2.01-2.74)** |

^a^ Estimates adjusted for age

Significant estimates (95% confidence level) are shown in bold type

**Table S4: Odds ratio (OR) estimates related to admission to intensive care unit (ICU) on entire population, stratified by gender and age group (one model for each variable)**

|  |  | **Male** | | **Female** | |
| --- | --- | --- | --- | --- | --- |
| **Variable** | **Category** | ***(N=370)* 45-59 ___________  OR (95% CI)^a^** | ***(N=748)* 60-74 ___________  OR (95% CI)^a^** | ***(N=133)* 45-59 ___________  OR (95% CI)^a^** | ***(N=257)* 60-74**  **___________  OR (95% CI)^a^** |
| **Charlson**  **Comorbidity  Index** | 0 | Ref | Ref | Ref | Ref |
|  | 1 | **1.69 (1.31-2.18)** | **1.45 (1.22-1.73)** | 1.29 (0.81-2.05) | **1.80 (1.33-2.43)** |
|  | 2-3 | **2.35 (1.58-3.47)** | **1.92 (1.58-2.35)** | **3.24 (1.88-5.60)** | **3.73 (2.70-5.15)** |
|  | 4+ | **3.95 (1.95-8.01)** | **3.58 (2.66-4.81)** | **2.71 (0.38-19.47)** | **9.36 (5.61-15.61)** |
| **Oncological**  **disease** | - | **2.20 (1.24-3.92)** | 1.25 (0.94-1.65) | 1.00 (0.32-3.13) | 1.39 (0.82-2.34) |
| **Cardiovascular disease** | - | **2.15 (1.56-2.97)** | **1.50 (1.25-1.80)** | **2.66 (1.47-4.83)** | **2.53 (1.84-3.47)** |
| **Respiratory**  **disease** | - | **3.00 (2.00-4.50)** | **1.97 (1.52-2.56)** | **2.77 (1.22-6.30)** | **4.69 (3.22-6.83)** |
| **Myocardial**  **infarction** | - | **2.25 (1.34-3.78)** | **1.62 (1.26-2.08)** | 1.76 (0.25-12.63) | **3.30 (1.92-5.67)** |
| **Heart failure** | - | - | **2.16 (1.33-3.50)** | - | **4.28 (1.76-10.39)** |
| **Cerebrovascular disease** | - | 2.09 (0.93-4.70) | **1.68 (1.20-2.36)** | 1.40 (0.19-10.01) | **2.78 (1.52-5.10)** |
| **Diabetes** | - | **1.99 (1.42-2.78)** | **2.19 (1.87-2.56)** | **3.51 (2.11-5.84)** | **3.19 (2.42-4.21)** |

^a^ Estimates adjusted for age

Significant estimates (95% confidence level) are shown in bold type

**Table S5: Odds ratio (OR) estimates related to death within 30 days from the first positive swab on entire population, stratified by gender and age group (one model for each variable)**

|  |  | **Male** | | **Female** | |
| --- | --- | --- | --- | --- | --- |
| **Variable** | **Category** | ***(N=97)* 45-59 ___________  OR (95% CI)^a^** | ***(N=461)* 60-74 ___________  OR (95% CI)^a^** | ***(N=23)* 45-59 ___________  OR (95% CI)^a^** | ***(N=168)* 60-74**  **___________  OR (95% CI)^a^** |
| **Charlson**  **Comorbidity  Index** | 0 | Ref | Ref | Ref | Ref |
|  | 1 | **2.00 (1.15-3.47)** | **1.67 (1.31-2.13)** | 2.72 (0.82-9.05) | **2.23 (1.51-3.29)** |
|  | 2-3 | **8.92 (5.27-15.11)** | **3.59 (2.84-4.54)** | **22.01 (8.45-57.33)** | **5.12 (3.44-7.61)** |
|  | 4+ | **28.98 (14.96-65.14)** | **8.19 (6.07-11.15)** | **59.56 (12.54 -272.78)** | **20.09 (12.25-32.96)** |
| **Oncological**  **disease** | - | **8.32 (4.42-15.66)** | **1.83 (1.36-2.45)** | **11.41 (4.22-30.81)** | **2.02 (1.19-3.43)** |
| **Cardiovascular disease** | - | **4.89 (3.03-7.89)** | **2.45 (2.00-2.99)** | **3.79 (1.12-12.80)** | **4.00 (2.87-5.59)** |
| **Respiratory**  **disease** | - | **7.53 (4.34-13.06)** | **3.95 (3.08-5.06)** | **8.53 (2.53-28.75)** | **8.42 (5.81-12.19)** |
| **Myocardial**  **infarction** | - | **3.43 (1.50-7.88)** | **2.16 (1.64-2.84)** | - | **4.07 (2.31-7.18)** |
| **Heart failure** | - | **6.98 (1.71-28.37)** | **3.03 (1.83-4.99)** | - | **11.06 (5.81-21.04)** |
| **Cerebrovascular disease** | - | **8.25 (3.60-18.92)** | **3.67 (2.72-4.94)** | - | **5.93 (3.58-9.82)** |
| **Diabetes** | - | **3.28 (1.91-5.64)** | **2.50 (2.06-3.04)** | **8.12 (3.20-20.65)** | **3.58 (2.59-4.94)** |

^a^ Estimates adjusted for age

Significant estimates (95% confidence level) are shown in bold type

**Supplementary Figure 1: Diagram of possible transitions among states**


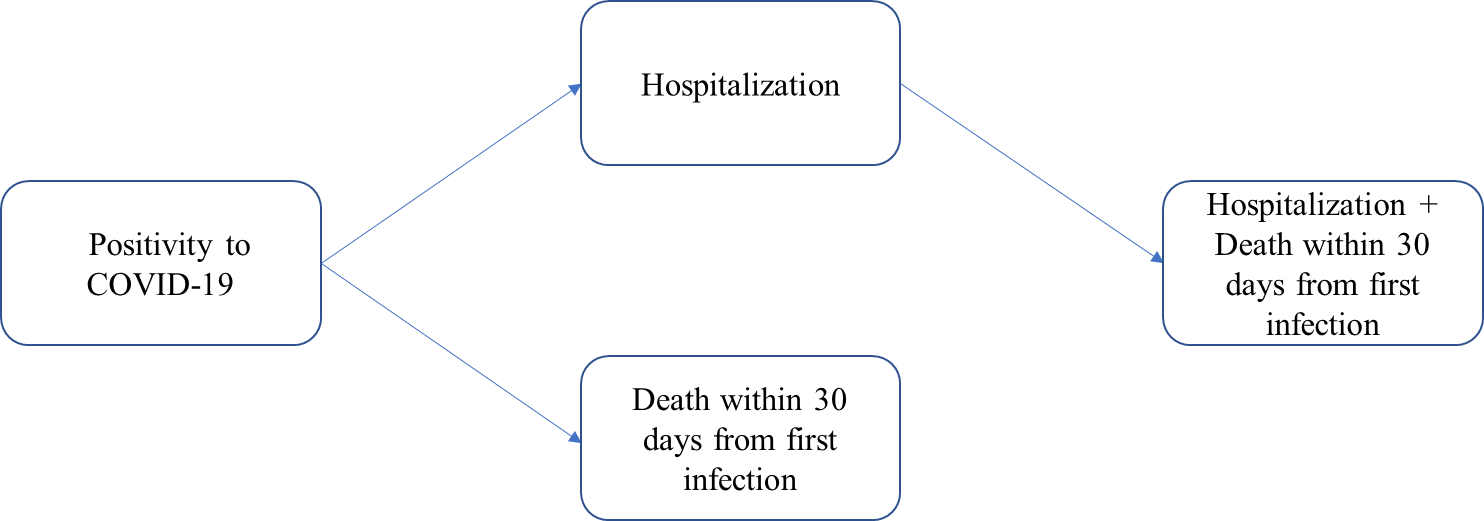


**Supplementary Figure 2: Probability of transitions among states related to multistate models adjusted for age and month of infection, stratifying by Charlson Comorbidity Index (CCI), gender, and age group**

1. **Males, 45-59 years old**

1. **Males, 60-74 years old**

1. **Females, 45-59 years old**

1. **Females, 60-74 years old**

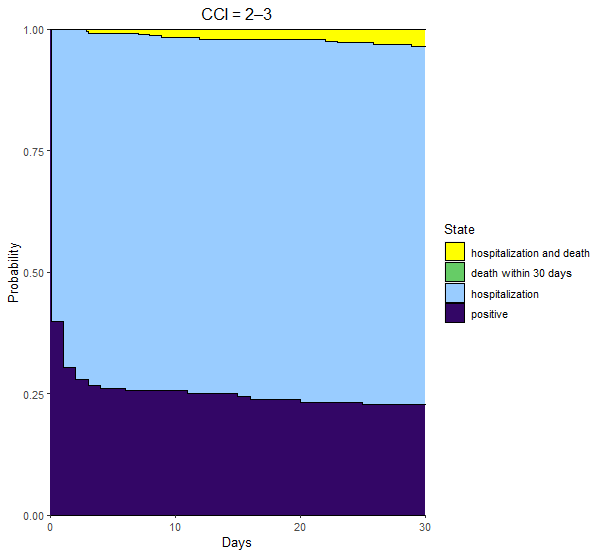

Supplement: Multimedia Appendix 1 [file publichealth_v9i1e41404_app1.docx]
